# Challenges in the Management of Care of Brain-Dead Patients in the Donation Process: A Qualitative Content Analysis

**Published:** 2020

**Authors:** H. YazdiMoghaddam, Z. S. Manzari, A. Heydari, E. Mohammadi

**Affiliations:** 1 *Iranian Research Center on Healthy Aging, Operating Room Department, Faculty of Paramedices, Sabzevar University of Medical Sciences, Sabzevar, Iran*; 2 *Faculty of Nursing and Midwifery, Mashhad University of Medical Sciences, Mashhad, Iran*; 3 *Department of MedicalSurgical Nursing, Faculty of Nursing and Midwifery, Mashhad University of Medical Sciences, Mashhad, Iran*; 4 *Evidence-Based Care Research Center, Department of Medical-Surgical Nursing, Faculty of Nursing and Midwifery, Mashhad University of Medical Sciences, Mashhad, Iran*; 5 *Department of Nursing, Faculty of Medical Sciences, Tarbiat Modares University, Tehran, Iran*

**Keywords:** Care, Brain dead, Donation process, Content analysis

## Abstract

**Background:**

In care of brain-dead patients, nurses face several challenges. It is important to determine the context behind these challenges since they affect the performance of nurses and the organ donation process.

**Objective:**

To identify factors affecting the emergence of challenges related to the management of brain-dead patients by nurses in the donation process.

**Methods:**

In this qualitative conventional content analysis, data were collected by performing 28 semi-structured and in-depth interviews with nurses working in the ICUs. Purposive sampling started from March 2014 until saturation, which was reached in June 2016. Data analysis occurred simultaneously with data collection.

**Results:**

Qualitative analysis of contents provided from interviews led to the extraction of themes that showed the experience of nurses about the challenges of caring for brain-dead patients in the donation process. These themes included “doubt and conflict in accepting the situation” and “defects in an effective and targeted care system.” In the end, the main theme of “inconsistency and incompatibility of care management” was abstracted.

**Conclusion:**

According to the results of the study, factors involved in the emergence of challenges for nurses in care management included defects in education or managerial problems, which increased tension for nurses.

## INTRODUCTION

Brain death was clinically defined in 1959 for the first time [1]. According to statistics, one of the main causes of brain death in Iran is traffic accidents [2]; 16,000 traffic-related deaths were reported in 2016 [3]. One out of every 10 accidents results in death, and one in every 100 deaths is brain dead. Compared to other countries, the highest rate of brain death belongs to Iran [2]. Given the high rates of brain death, nurses have the most experience of brain death care [4]. As such, their role in the early detection and proper care of brain-dead patients, which also has an important impact on the process of donation, is more highlighted [5]. 

Brain death management has various challenging aspects due to its complicated nature, causing difficulties for nurses and affecting the quality of care of organ donors. One of these challenges is understanding the concept of brain death. While doubts about brain death diagnosis must be essentially observed outside intensive care unit (ICU), some ICU nurses have doubts in a way that they believe that a brain-dead patient is still alive [6]. In a study conducted by Keshtkaran, nurses were involved in a type of ambiguity and doubt in care-giving to the potential organ donors. Such confusion complicates providing care to potential donors by nurses and strengthens the denial of patients’ family [7].

Smith found that nurses had fear in facing organ donors because of their doubt and uncertainty about their death due to their lack of awareness of brain death diagnostic tests and the process [8]. Despite physiological knowledge about brain death, the experience of caring for these patients is stressful to nurses, even after a long time since understanding the physiology of death, and is in conflict with the appearance, heat, and heart rate of the patient. This increases the stress of nurses and leads to problems in understanding the diagnosis of brain death [4]. Other studies also found that nurses are uncertain about the concept and diagnosis of brain death [6, 9, 10] due to their insufficient knowledge about diagnostic tests for brain death and the confirmation process [11]. This complicates understanding the concept of brain death by nurses and affects the management of care and the donation process.

The cultural and ethical aspects of brain death and organ donation are other challenging dimensions of this process. Despite the advancement of knowledge and technology, no one can fully assure that the clinical diagnosis made is correct and that the brain is completely dead [12]. Mattiussi found that the presence of some weak movements associated with spinal reflexes may increase the ambiguity [13]. Therefore, nurses doubt the nature of the patient’s death and life, which complicates accepting brain death [12]. Kim showed that individuals’ attitudes, beliefs and behaviors are influenced by their cultural background and religious beliefs [14].

One of the most important moral challenges is confirming the death of a person with vital signs. Studies have demonstrated that nurses have expressed concerns about brain death and organ donation in a cultural and religious context [15, 16]. Altınörs found that there is no consensus among the experts of the field regarding the concept of brain death. Generally, the fear of hasty defining of death and lack of its discrimination from a coma is the main reason for such doubts and lack of consensus in this respect [12]. Detection of the time of death is also an important moral challenge. The moral challenge roots from that different religions and cultures cannot easily accept that a person who has vital signs would be dead [17]. In a study by Jeon, it was indicated that despite the approval of the donation law in Korea in 2000, nurses have a passive attitude toward organ donation due to cultural reasons and lack of sufficient knowledge [18]. These beliefs have impeded progress in detecting brain death due to insufficient knowledge of nurses and health care team about brain death and organ donation [19], which can lead to prolongation of the patient care process and negatively affect the quality of care for organ donors [20, 21]. Considering the centrality of this issue, the attitude of nurses toward brain death is thus significantly important in any society.

The care of potential organs of brain-dead patients is one of the issues emphasized by nurses in the care process. In a study conducted by Foresberg, it was reported that intensive technical, and medical and nursing interventions are required after the announcement of brain death in order to make the donation of organs possible [22]. Various studies have also indicated the low level of knowledge of nurses about brain death and organ donation [18, 23-25]. Consequently, prolongation of this process can lead to death or loss of vital organs due to the specific and vulnerable conditions of the organ donor [26]. According to Siddiqui, it is better for team care to attend a training program regarding their concerns before implementing a deceased-donor organ transplant system in hospitals to increase deceased-donor consent rates [15]. Research revealed that providing special educational programs on care process to nurses is required to be commenced from university level and continued in the form of in-service training for increasing knowledge about the process of brain death and organ donation [27-29]. 

Providing care to potential donors by nurses is a complex process and requires considerable mental preparation and skills, which are the weak areas among nurses today. In this respect, nurses do not often accept giving care to brain-dead patients due to lack of accepting their condition. However, this refusal is not because of intentional negligence about the care by nurses and is mostly related to insufficient information and lack of mental preparedness in accepting this situation, which affects the organ donation process [30]. In a study by Salehi, it was found that the care of potential organ donors was a difficult experience for nurses and could be a threat to nursing health and quality of care [31]. 

Considering the importance of preserving the health of organs, it is necessary that care management be carried out with high accuracy and precision. With regard to the challenges presented above, nurses are faced with problems in the care process that affect the donation process. In order to achieve this goal, paying close attention to the causes of the challenges in this field by considering the actual experience of nurses in this situation is of paramount importance. This is mainly due to the fact that no proper planning could be made for making the right decision regarding complicated care of these patients as long as the different aspects of the experience of nurses in this process are not discovered and identified. It is possible that complications caused by the involvement of nurses in the care process of these patients also affect their other roles in providing care to other patients [32, 33]. Therefore, considering the nature of qualitative studies, we can conduct an in-depth study on the nurses’ mental status about the health care challenges experienced and take effective measures to improve the quality of care provided to organ donors by proper planning and policy-making. With this background in mind, this study aimed to identify the underlying factors and challenges associated with the management of brain-dead patients.

## MATERIALS AND METHODS

Regarding the aim of the study, quality approaches that seek to understand the emotional and human meanings hidden in their daily experience of life were used. Since the goal of the study was obtaining a deeper understanding of the factors affecting the challenges faced by nurses in care of brain-dead patients, the qualitative content analysis method was applied [34]. Participants included nurses working in the ICU with a history of caring for brain-dead patients and a minimum of one year of experience in ICU. Other inclusion criteria were lack of history of depression or psychiatric disorders and willingness to present their experience.

In this study, the main data collecting method was a semi-structured interview with open and face-to-face questions. First, the researcher explained the research objectives and obtained informed written consent from each participant. Afterwards, the interviews were initiated by asking open questions. All interviews were recorded and transcribed verbatim. The main questions of the interview were “could you please explain about the time you must provide care to a brain-dead patient?,” “Can you describe each care situation in detail?,” and “what did you feel in that situation?” In addition, other questions such as “can you explain any possible experience you have had in this area?” Were asked to guide the interview based on their dialogues. The duration of the interview varied between 45 and 90 minutes. 

By progressing the research, interviews were guided based on questions asked, and the researcher directed his questions based on important categories created [35]. Sampling was carried out until saturation of data purposively, meaning that sampling continued until lack of receiving new codes in final interviews and completion of all conceptual levels [36]. In this regard, 25 interviews and three complementary interviews were made with participants. 

Ethical approvals were obtained from the Ethics Committee of Mashhad University of Medical Sciences. In addition, informed written consent was obtained prior to the research, and subjects were ensured of the confidentiality terms regarding their personal information. 

Data analysis was performed using qualitative content analysis and a conventional approach, which is mainly used in an inductive way and in qualitative studies with the purpose of providing an in-depth description of the phenomenon when the existing theory or studies on the phenomenon are limited [37, 38]. 

To this end, the texts of the interviews were reviewed several times and the irrelevant statements were excluded. Afterwards, the data were broken down into semantic units (codes) in the form of sentences and paragraphs related to the original meaning. Following that, the appropriate codes were written for each semantic unit, and the codes were categorized based on conceptual and semantic similarities. There was a downward trend in the reduction of data in all units of analysis and main and subclasses. In the next stage, the codes were placed in the main categories, which were more general and conceptual. Finally, the themes were abstracted, and the analysis process was modified by adding each repetition interview and classes [39].

In order to ensure the credibility of the data, a friendly and precise relationship was established with the participants to collect more information. For the adequacy of the validity of the findings, the manuscripts and the analysis units along with the primary codes extracted were provided to the participants, followed by receiving their comments and making the necessary modifications. Moreover, interviews and initial coding and themes were reviewed by several professors in the field of qualitative research. Sampling with maximum variance, meaning interviews with different people in terms of age, sex, work experience, workplace, increased the acceptance, confirmation, and transmissibility of the data. The allocation of sufficient time to the study was another factor involved in increased credibility of the data. The validity of the results was followed up for several months by repeated and long-term analysis [37].

## RESULTS

In this qualitative research, 28 interviews were made with nurses. The participants were working in hospitals affiliated to Mashhad, Neyshabour, and Sabzevar Universities of Medical Sciences. Moreover, these individuals were within the age range of 28–49 years and had work experience of 4–26 years. Analysis of data led to the identification of 1237 primary codes, 35 subclasses, four categories and two themes (Table 1).

**Table 1:** List of themes and categories for the central theme of “inconsistency and incompatibility of care management”

Theme	Category	Subcategory
Defects in efficient and targeted care system	Defects in efficient organizational management	Unspecified and non-purposeful division of labor
		Undesirable supervision in care
		Lack of planning targeted and effective training
	Inefficient and non-targeted care system	Ineffective care following insufficient experience
		Ineffective care following insufficient knowledge
		Lack of skill in proper care of the family
		Incompetence of Caregiver
Hesitation and conflict in accepting the situation	Doubts and conflicts between doctors and nurses in understanding treatment and care	Disagreement between physician and nurse in treatment-care processes
		Doubt about brain death diagnosis
	Duality of emotions and feelings about organ donation	Spiritual and religious acceptance vs. lack of acceptance of donation
		Emotional acceptance/lack of acceptance of donation

Theme 1: Defects in Efficient and Targeted Care System 

One of the factors that make the management of brain-dead patients in the ICU stressful for nurses is defects in the care system. Despite the importance of caring for the donation candidates and emphasizing the increased number of donations, the care system has designed no measures to improve the quality of care and preserve the potential organs for donation. These issues expose nurses to the stress of keeping the candidate alive and fear of failure. This theme was extracted from the following two categories.

A: Defects in Efficient Organizational Management 

This concept has three subcategories and reflects the challenging conditions faced by nurses in the management of brain-dead patients, in a way that while most of the nurses in the present study tended to provide proper care of candidate patients, they were faced with several problems due to the management conditions governing the care system. One of the management problems is related to the division of labor. In this regard, despite the importance of preserving the health of potential organs, there are some problems in the management of care of these patients due to the conditions prevailing in the ICU.

A1: Unspecified and Non-purposeful Division of Labor 

Nurses face many challenges, such as increased work pressure and the difficulty of care to keep the patient alive for donation, and experience stressful situations because they must provide precise care to patients to avoid damage to potential organs for donation. Nevertheless, the low use of care of brain-dead patients continues to dominate the division of labor in ICUs. “In ICU, no attention is paid to the status of patients (whether they are in an acute condition or are donation candidates). It is a common rule that they assign two patients to a nurse simultaneously and do not care about the result. They just divide the labor. As a nurse, you must care for both patients, one of whom is young and the other one is an organ donor. It means that you are constantly stressed about your patients until the end of the shift.” (Participant [P] 13) 

In the division of labor, no attention is paid to patients whose family members have not given consent to organ donation despite being a candidate. This attitude reduces the attention and sensitivity of patient care unconsciously even though the patient can be a donation candidate, leading to the neglecting of many care procedures of patients and thereby creating many problems for nurses. 

“Division of labor is carried out in a way that caring for brain-dead patients who can be a donation candidate but their families have not given consent is not important. They assigned a brain-dead patient and a critically ill patient to me. The care of latter was so time-consuming that I did not have time to care for the brain-dead patient. I feel guilty about this issue since the family of the brain-dead patient might come forward on the last minute and give consent to donate the organs of their patient but it would not be useful due to lack of providing proper care to the patient.” (P27)

A2: Undesirable Supervision in Care

This concept reflects the conditions that complicate the work of nurses in a way that simultaneous assigning of a critically-ill patient and a brain-dead patient to nurses expose them to high work pressure and physical fatigue and might even lead to improper patient care in some cases. As such, the lack of supervision and control over the provision of nursing cares results in undesirable care far from the care standards or care without a scientific view of the patient, which causes problems in the care of the candidates.

“None of the ICU nurses ask about the condition of the patient and whether there has been a change in the patient’s device mood or not. The same rate is observed on the device screen. Therefore, it becomes a routine, and the status of patients is not scientifically evaluated. Even lack of supervision of candidate patients has led to problems in the condition of patients and lack of ability to donate the organs.” (P3)

“No one controls the condition of patients and there is no accurate supervision to determine whether proper care is provided or not. One time, I was really tired and did not have the strength to suction the candidate. Ultimately, I did not suction the patient but felt bad and guilty about it.” (P1)

A3: Lack of Planning Targeted and Effective Training

Nurses require adequate information on detection of brain death (e.g., knowledge of spinal reflexes) in the process of comprehensive care of candidate patients for preserving the organs. These issues are more evident in novice nurses with less work experience and lower level of knowledge, intensifying their concern about keeping the patient healthy until donation and fear of failure. This issue causes severe care tensions in nurses in a way that lack of knowledge about spinal reflux leads to doubt about the diagnosis and increases the possibility of slacking. 

“I did not know about this issue at first. I would often see that the toes or head of a patient moved, so how can he/she be brain dead? I would doubt the diagnosis, which would reduce my focus on the care of these patients…Now, I know that all of these are due to spinal reflex and that is why I know that I was lacking…” (P11)

Training defects are not just about patient care and involve the care of the family of candidate patients, decisions of whom affect the possibility of donation. Nurses are afraid of giving false information to families since inaccurate information about the diagnosis of brain death might consequently lead to lack of consent for organ donation. 

“When the families ask a question about their patients, I am always afraid to give false information, which could lead to their doubt about the diagnosis of their patients and lack of consent for donation.” (P15) 

Failure to provide training based on needs assessment causes ICU nurses, who deal with brain-dead patients, to be incapable of providing proper care to the patient, and be afraid of failure in spite of dedicating significant efforts to the care process.

“They just focus on statistics and provide no training to nurses regarding how to care for this type of patients…Since nobody provides scientific educations in this regard, I always think that I have made a mistake and something might happen in the donation process until the end of the donation procedure.” (P7)

B: Inefficient and Non-targeted Care System

Care of brain-dead patients is important in the health system due to the need for preserving the donation organs. Factors such as inadequate knowledge, experience, and communication skills of nurses have caused some challenges in the management of brain-dead patients and are associated with a lack of accurate care in this regard. In addition, they increase the tension of keeping the patient alive for donation in nurses, who are concerned with possible defects in this area.

B1: Ineffective Care Following Insufficient Experience

Care knowledge integrated with work experience is one of the most important issues in the process of patient care, in a way that nurses are incapable of proper management of possible donors and experience some challenges due to lack of sufficient experience in this regard. 

“Experience is significantly crucial in care of candidates. In the beginning of my work, I was often behind in my tasks due to lack of sufficient experience. For example, I would become concerned if there was an issue in the tests of my patient. I would not know what to do and whether to inform the ward physician or the donation committee physician…I was constantly worried and would think that the patient will lose his/her kidneys.” (P1)

B2: Ineffective Care Following Insufficient Knowledge

Having insufficient knowledge about care tasks leads to nurses’ uncertainty about finishing the donation process, in a way that they are skeptical about the care and experience a great deal of stress in patient care management. 

“They told me that this is a candidate and must be kept alive. I was really stressed and was worried about my work and the possibility of death of the patient before donation. I was not trained for this type of care and my mind was constantly preoccupied with this issue…” (P13)

B3: Lack of Skill in Proper Care of the Family

In the care process, in addition to patient care, care of the family of the candidate is important as well. However, due to the inability to communicate and help relieve the family, nurses are unable to provide emotional support to the candidate’s family, and therefore, experience extreme tensions. 

“It is upsetting when you lack the necessary knowledge and skill to communicate with them and reduce their sadness and make them calm. This makes you feel incapable.” (P12)

B4: Incompetence of Caregiver 

The inability of nurses in terms of scientific and skill management of care leads to lack of proper care of the candidates and inability to keep the patient alive for donation despite the efforts to obtain family consent. This issue creates a sense of failure in nurses. 

“I have insufficient information about the care of this type of patients, which creates extreme stress for me due to lack of ability to keep the patient alive. Sometimes, insufficient knowledge in this area leads to the loss of the patient by neglecting a suction…” (P28)

Theme 2: Hesitation and Conflict in Accepting the Situation 

In terms of dealing with brain death, nurses experience conflicts in confirmation of diagnosis and transfer to the transplantation center and even after donation. Conflicts between the doctors and the nurses after having doubts about the diagnosis of brain death and various aspects of donation exposes nurses to a great deal of stress, in a way that some nurses experience mental preoccupation and fear of failure. This theme has two categories, presented below. 

A: Doubts and Conflicts between Doctors and Nurses in Understanding Treatment and Care 

This concept has two subcategories and expresses that despite the importance of cooperation between health care team members, nurses feel uncomfortable about the conflicts between themselves and doctors in the process to care for non-candidate brain-dead patients, facing challenges in this regard. 

A1: Disagreement between Physician and Nurse in Treatment-Care Processes

The lack of training of nurses about the concept of brain death and the diagnostic confirmation process creates doubts in some of these individuals to accept the doctor’s confirmation, making it difficult for them to accept the brain death of patients. Therefore, they are in conflict with the doctor regarding the continued care of non-candidate patients.

“Doctors are not that eager to keep non-candidate patients alive. They advise against resuscitation of these patients since they believe that other patients in critical conditions might need a bed…I feel terrible if a patient has developed cyanosis and I do nothing about it.” (P18)

However, nurses are not willing to refuse providing care to these patients and discontinuing medications for non-candidate patients due to work ethics. This is mainly due to the fact that nurses are not sure about the diagnosis and are afraid of guilt because of failure in this regard. 

“If the patient has no condition for donation, my conscience does not allow me to stop the administration of dopamine and other medications of the patient. I would feel guilty if I do not provide care to the patient. What if the diagnosis was incorrect?” (P1)

A2: Doubt about Brain Death Diagnosis 

Some nurses are still uncertain about the diagnosis of brain death and early donation of the patient’s organs, which causes doubt in the diagnosis of brain death by doctors.

“The first issue is determining whether the diagnosis is accurate or not and the patient is definitely brain-dead and his/her organs can be donated.” (P10)

The lack of nurses’ knowledge about the state of brain death causes doubt about the brain death of patients following the continuous observing of apparent signs of life in these individuals. 

“Yes, I believe the patient is dead since the brain has no function. However, the patient is not dead since the heart is still working. The patient is dead when the heart does not work anymore.” (P12)

B: Duality of Emotions and Feelings about Organ Donation

Importance of organ donation has led to the distinguishing of care of brain-dead patients from other patients in ICU. Nevertheless, some nurses still have doubts and are uncertain about rejecting or accepting the issue emotionally. 

Lack of training of nurses in terms of legal and religious issues related to donation exposes them to mental conflicts and fear of punishment due to being involved in the process of donation. This concept has the following subcategories. 

B1: Spiritual and Religious Acceptance vs. Lack of Acceptance of Donation

Nurses accept donation and attempt to keep the patient alive for donation based on their beliefs. However, some nurses do not accept the nature of donation due to their spiritual imaginations and doubts about the donation process.

“Is donation a right choice or not? Is it accepted by all authorities? I am still not sure about this issue since I believe that brain-dead patients are still alive and I feel guilty when their organs are donated.” (P6)

The doubt of some nurses about the donation is because they think that in a religious sense, a person is still alive as long as there is a spirit in the body. Therefore, this problem makes nurses uncertain about religious and scientific aspects of donation. 

“I always think that when the kidney of the patient is removed, his/her heart is still working. There is still a spirit in the body and the patient is still alive, right? However, scientifically, the patient is dead and there is nothing we can do for him/her. Therefore, the body organs are donated…” (P9)

B2: Emotional Acceptance/Lack of Acceptance of Donation

Accepting the donation of organs of a brain-dead patient by the family makes nurses happy, and their belief in the rescue of patients in need of transplantation leads to the acceptance of donation and dedication of extensive efforts to care of donation candidates.

“There was a donation candidate, for whom I provided care and followed up the tasks relevant to the patient. I made great efforts to keep him alive since he had a critical condition. I was really happy when I heard that his organs were donated to four patients…” (P15)

On the other hand, some nurses are uncertain about donation since they believe that patients are suffering during the donation process and their lives are taken before their actual death.

“This is true that a person is cut into several pieces and each of his organs is donated to another patient…I feel bad when the heart is donated since the life of the patient is taken by doing so…” (P12)

Nurses are faced with challenges during caring for brain-dead patients, which not only increase the stress level of these individuals in the patient care process but also affect the donation procedure. Therefore, proper management is required to deal with the possible challenges in this regard.

## DISCUSSION

The present study aimed to identify the influential factors in the emergence of the challenges associated with the care and management of brain-dead patients. The most important underlying issues that could cause challenges for nurses are “doubt and conflict in the acceptance of the situation” and “defects in the efficient and targeted healthcare system.” Despite these problems, nurses carry out the complete care of patients with a diagnosis of brain death until the last minute. According to the results of the present study, the “inconsistency and incompatibility of health care management” was the most effective concept demonstrating the major influential factors in the emergence of these challenges. Therefore, this concept could be considered the central theme covering the other themes of the current research. 

Although the components and dimensions of the concepts creating these challenges were determined in this review study, none was considered an independent underlying concept. Therefore, we have provided a complete summary of these factors compared to the other studies in this regard. The theme “defects in the efficient and targeted health care system” was introduced as one of the main underlying factors with respect to the challenges faced by nurses in this area. In this regard, Folden evaluated the experiences of Swedish nurses about organ donation, proposing the concepts of “lack of leadership,” “sensitive role of physicians,” and “absence of an organizational structure.” In the mentioned study, nurses claimed that the lack of leadership resulted in doubt and feeling of abandonment following the care of candidate patients. In addition, they considered the lack of an organizational structure for organ donation a major limiting factor in the care process of these patients [9]. According to Irodat statistics (2015), there has been an increase in the rate of organ donation in Sweden [40], indicating the correction of organizational structures in this area from 2011 onwards. 

In Iran, 5000–8000 brain-dead cases were reported in 2016, 2500–4000 of whom were identified as potential donors. Moreover, 7–10 patients die on the waiting list daily. These statistics reveal the importance of timely identification and proper care by the health care team, especially nurses [3]. However, the results of the present study demonstrated the defective structure and organizational management governing the health care system. As a result, nurses are still faced with numerous problems and complications in the management of patients with brain death diagnosis, and these challenges are intensified due to the issues in the health care system and lack of organizational support in this regard. 

According to the literature, nurses consider the lack of support by their supervisor a key influential factor in the challenges associated with the care of potential donors [9]. Since nurses experience high stress levels in the care of brain-dead patients, they need the support of the health care system. This is mainly due to the fact that lack of support by supervisors and health care system increases the stress level of these health care professionals [19]. For instance, Foresberg claimed that nurses had emotional needs in the care of brain-dead patients. In other words, nurses needed the support of the health care management in the care of organ donors [22]. In another study, Karimi reported that nurses experienced severe issues in the care of dying patients. In this regard, nurses require organizational support, and not meeting the needs of these professionals inadvertently affects these professionals and patients [41]. 

In general, nurses experience the highest stress level in care processes due to the special condition of ICU [42]; the management of dying patients exposes nurses to high occupational stress levels [43]. As such, it seems crucial to provide training and support to ICU nurses [44]. The support conditions in the work environment positively influences the ability of nurses to use their knowledge and skills at work [45], so that a basic understanding of the care of organ donors leads to the development of proper strategies to improve the quality of care and success in organ donation, while addressing the need to provide educational and support services to nurses [12]. Meanwhile, most of the studies conducted in this regard in Iran and foreign countries, such as the studies by Kima, Mahdiyoun, and Azmandian, have denoted the low knowledge level of nurses about brain death and organ donation process due to inadequate training in this regard [18, 23, 24, 46]. 

The sensitivity of care for organ donors and the associated stressful conditions, along with the lack of knowledge of nurses exposes them to several psychological problems [46, 48]. According to Salehi, there were scientific knowledge defects in the care of candidates for the preservation of organs for donation, which increases the stress of nurses. These defects could be attributed to the lack of adequate knowledge of health care staff regarding the donation process during nursing education in nursing schools. According to nurses, the knowledge required to manage an organ donation process is mostly acquired through the experience of other colleagues, physicians, and transplantation team [31]. This is consistent with the “lack of planning in the provision of targeted training” as one of the themes in the present study. Considering the similarity of the care structure in both studies, it seems necessary to design a supportive model in line with the educational needs of nurses in this regard. 

According to the findings of Lemes, the reaction of the nursing team to brain death represents their fear and anxiety, so that the care of organ donors is difficult for these individuals and leads to their reduced attention in their care [16]. Furthermore, previous studies have indicated that a nurse providing care to an organ donor should have the rational and emotional knowledge of brain death, as well as the proper attitude toward this issue [9]. In the current research, the theme of “doubt and conflict in the acceptance of the situation” was considered another challenge for nurses. Disagreement and conflict are among the natural components of any relationship and are inevitable in health care systems, such as hospitals [49]. In this regard, Keshtkaran extracted the category of “doubt about the diagnosis and confirmation of brain death” in the evaluation of the lived experience of nurses in the care of organ donors, stating that in the viewpoint of nurses, knowledge and technology are still relative despite their advancement. In other words, no one can be completely sure about clinical diagnoses. Nurses do not know whether a brain-dead patient is considered dead or alive, which is mainly due to their uncertainty about the nature of the death and life of the patient [7].

On the same note, Pearson evaluated the experiences of nurses about the concept of ambiguity in the diagnosis of brain death, realizing that since the physical condition of brain-dead patients is similar to other patients and the nurses providing care to these individuals are also similar to the nurses of other patients based on their professional and ethical obligations, these issues increase uncertainty about accepting the occurrence of brain death [50]. Studies by other researchers have also indicated the category of ambiguity and uncertainty about the diagnosis of brain death on behalf of physicians [7, 51-53], which leads to high stress levels in nurses.

Another challenge in the care management of brain-dead patient is the duality of emotions and feelings about organ donation. In this regard, Miller marked that nurses had fear when faced with organ donors due to uncertainty about their death. This was mainly due to the fact that they lacked adequate knowledge about brain death diagnosis and its confirmation process [54]. Studies in this regard have also shown that the main concerns regarding issues (e.g., diagnostic tests of brain death and mindsets about donation) could cause anxiety and doubt in ICU nurses, where the highest number of donors is identified [55]. The results of another research demonstrated that the main concern of nurses in the care of organ donors and their families is to maintain the organs of brain-dead patients. Nevertheless, the experiences of nurses have revealed conflicting meanings, which are mainly due to ambiguity in the declaration of the death of the patients who still have their vital signs [50]. For instance, the appearance of the patient (e.g., warmth and heart rate) is perceived as a vital sign. On the other hand, the provision of care to organ donors is carried out for organ donation, which makes it more difficult for nurses to accept the death of a brain-dead patient. 

In the study by Floden, the theme of “different attitudes toward organ donation” emerged in an attempt to describe the experiences of nurses in the care of organ donation candidates. This theme encompassed specific perspectives, such as the fact that the responsibility of nurses is to provide care to a living person and not a dead individual and the fact that the entire process is unpleasant, so that uncertainty about the brain death of an individual who is still alive is tangible in ICU nurses due to their inadequate knowledge in this respect [6].

Cohen argued that confusion and ambiguity in the verification of brain death and differences in perspectives on the concept of death might be associated with some consequences in the process of care provision to organ donors [56]. Furthermore, Rittner believes that ambiguity and doubt in nurses could be due to professional ethical challenges since nurses provide care to the patients who seem to be alive, but are in fact dead, and donation of some of their organs could save the lives of others. These issues cause ethical challenges for nurses. In fact, religious and cultural beliefs and moral and emotional conflicts overcome the scientific and practical training of nurses, thereby preventing the complete and clear acceptance of the death of a brain-dead patient [57]. While communities have different medical conditions in terms of religion and culture, nursing systems are faced with the challenge of uncertainty and conflicts about brain death and organ donation, which persists until today.

The themes in the present study represented the factors or conditions that could give rise to numerous challenges and ambiguities in the nursing care provided to brain-dead patients, which affect the performance of nurses in the care of organ donors. Moreover, these issue cause limitations in the management of brain-dead patients with special needs. Meanwhile, transplantation statistics are still low in Iran due to the number of brain deaths, and maintaining the health of organs for transplantation is crucial. Therefore, despite the fact that nurses must be able to manage care to preserve organs, the mentioned factors impede the adoption of effective care and even disrupt the donation process by threatening the health of organs for donation or losing the organ donor in some cases due to mismatches. 

This was one of the first qualitative studies in Iran to focus on the context and conditions leading to the main concern of nurses in the management of brain-dead patients (candidates and non-candidates) based on their profound experiences. Considering the role of nurses in the quality of care of brain-dead patients and preserving the organs that are to be donated, health care policymakers could use our findings and consider the conditions to make the necessary planning to improve the quality of brain death care and provide the crucial support for nurses. 

One of the limitations of the current research was that the participants were only nurses employed in the hospitals affiliated to the medical universities in these cities of this province, who were willing to be interviewed; this issue does not reduce the importance of the role of the nurses in these process. Therefore, it is suggested that further investigations in this regard be conducted to identify various dimensions of the management of brain death care.
